# Incorporation of Graphene Nanoplatelets into Fiber-Reinforced Polymer Composites in the Presence of Highly Branched Waterborne Polyurethanes

**DOI:** 10.3390/polym16060828

**Published:** 2024-03-16

**Authors:** Ayşe Durmuş-Sayar, Murat Tansan, Tuğçe Çinko-Çoban, Dilay Serttan, Bekir Dizman, Mehmet Yildiz, Serkan Ünal

**Affiliations:** 1 Integrated Manufacturing Technologies Research and Application Center & Composite Technologies Centre of Excellence, Sabanci University, Teknopark Istanbul, Pendik, Istanbul 34906, Turkey; aysedurmus@sabanciuniv.edu (A.D.-S.); bekirdizman@sabanciuniv.edu (B.D.); meyildiz@sabanciuniv.edu (M.Y.); 2 Faculty of Engineering and Natural Sciences, Sabanci University, Tuzla, Istanbul 34956, Turkey

**Keywords:** highly branched functional waterborne polyurethane, carbon fiber-reinforced composites, graphene nanoplatelets, fiber sizing, interfacial properties, ultrasonic spray coating

## Abstract

Enhancing interfacial interactions in fiber-reinforced polymer composites (FRPCs) is crucial for improving their mechanical properties. This can be achieved through the incorporation of nanomaterials or chemically functional agents into FRPCs. This study reports the tailoring of the fiber–matrix interface in FRPCs using non-functionalized graphene nanoplatelets (GNPs) in combination with a waterborne, highly branched, multi-functional polyurethane dispersion (HBPUD). A unique ultrasonic spray deposition technique was utilized to deposit aqueous mixtures of GNP/HBPUDs onto the surfaces of carbon fiber fabrics, which were used to prepare epoxy-prepreg sheets and corresponding FRPC laminates. The influence of the polyurethane (PU) and GNP content and their ratio at the fiber–matrix interface on the tensile properties of resulting high-performance composites was systematically investigated using stress–strain analysis of the produced FRPC plates and SEM analysis of their fractured surfaces. A synergistic stiffening and toughening effect was observed when as low as 20 to 30 mg of GNPs was deposited per square meter of each side of the carbon fiber fabrics in the presence of the multi-functional PU layer. This resulted in a significant improvement in the tensile strength from 908 to 1022 MPa, while maintaining or slightly improving the initial Young’s modulus from approximately 63 to 66 MPa.

## 1. Introduction

Carbon fiber-reinforced polymer composites (CFRPCs) continue to replace traditional materials due to their distinctive features such as high strength, stiffness, and long service life for lightweight structural composites [1,2,3]. CFRPCs are increasingly used in aerospace, automotive, electronics, and other applications. Interfacial properties between the fiber and polymer matrix are an important factor determining the performance of CFRPCs in both thermosetting and thermoplastic resin-based systems [4]. Typically, while the surface of the virgin fiber is non-polar, the polymer matrix in CFRPCs tends to have a polar character. This inherent difference in polarity necessitates the enhancement of the naturally weak interfacial interaction between the fiber and the matrix to meet the performance requirements expected from composite materials. The effectiveness of load transfer is often ascribed to the interaction between the fiber and the matrix. If this interaction is too weak, stress transfer becomes limited in composite structures, leading to a compromise in performance. Consequently, poor interfacial adhesion diminishes the magnitude of load transfer between the matrix and fibers. Conversely, when the interfacial interaction intensifies significantly, cracks tend to propagate diagonally in the matrix, breaking the fibers. Striking a balance in interfacial adhesion is crucial as overly weak or overly strong interactions can adversely impact the load transfer phenomenon and, consequently, the overall structural integrity of the composite material. Achieving an optimal level of interfacial interaction is imperative for maximizing the performance and mechanical properties of CFRPCs. As a result, the stress concentration tends to be higher around these breakages [5,6,7]. The load-carrying capacity of composite materials hinges predominantly on the nature of the fiber–matrix bonding, encompassing both chemical and frictional interactions [8,9]. Extensive studies in the literature have elucidated diverse methodologies for the surface modification of carbon fibers, including wet chemical or electrochemical treatments, polymer coating, and plasma treatment [10,11,12,13,14,15,16,17]. These techniques aim to introduce various functional groups onto the carbon fiber surface, fostering robust adhesion between the fiber and the matrix. Through such modifications, researchers strive to optimize the interface, ensuring enhanced compatibility and, consequently, bolstering the composite material’s overall mechanical performance.

Carbon fibers inherently exhibit brittleness and low elongation, resulting in challenges such as yarn breakage and fluffiness during the manufacturing of CFRPCs. This inherent fragility necessitates surface treatment interventions. An effective sizing component becomes crucial, not only for enhancing chemical interactions between the fiber and the matrix to elevate interfacial adhesion properties but also for improving fiber bundling and overall performance characteristics [18]. Various functional groups, such as alcohol, carbonyl, and carboxylic acid, can be strategically incorporated onto the fiber surface through diverse sizing methods [19]. Furthermore, sizing facilitates the modification of the surface free energy of carbon fibers, thereby refining the interfacial features of composites. This, in turn, contributes to heightened mechanical characteristics in comparison with composites produced with untreated carbon fibers. 

In contemporary fiber sizing applications, waterborne polyurethane dispersions (PUDs) have garnered significant attention owing to their exceptional coating behavior and multi-functionality. PUDs also stand out for their environmental friendliness, non-toxicity, low viscosity, and remarkable adhesion capabilities with diverse polymeric matrices in composites [20,21,22]. An especially attractive feature is their ability to establish robust adhesion without requiring pretreatment of the fibers. This not only simplifies the sizing process but also aligns with environmentally conscious practices, making PUDs a compelling choice in fiber sizing for their versatile and eco-friendly attributes. PUDs emerge as highly suitable sizing agents also for carbon fibers in CFRPCs [23]. This suitability is attributed to their inherent polarity, marked by an ability to form effective bonds with the carbon fiber surface. Furthermore, the high elasticity and ductility of polyurethanes present an advantageous combination, offering the flexibility to tailor these properties based on the specific hard and soft segment structures within the backbone. This tailoring capability enables one to match the requirements of the carbon fiber reinforcement, contributing to enhanced compatibility and overall performance in CFRPCs [24,25]. Zhang et al. [26] previously documented that the treatment of carbon fibers with PUDs resulted in an elevation of surface energy. This increase was attributed to the introduction of nitrogen (N) atoms on the fiber surface through the treatment with PUDs. Consequently, the carbon fibers exhibited heightened wettability when combined with epoxy resin in CFRPCs. Such enhanced wettability is a key factor in promoting a more effective and intimate bonding between the carbon fibers and the epoxy resin matrix, contributing to improved overall performance in the resulting composite materials. Li et al. [27] conducted a study wherein waterborne PUDs based on a tartaric acid polyol were synthesized specifically for carbon fiber sizing. CFRPCs incorporating carbon fibers sized with PUDs demonstrated a remarkable improvement, exhibiting a 14.3% increase in tensile strength, a 24.4% increase in flexural strength, and an impressive 119.6% increase in impact strength when compared with CFRPCs from pristine carbon fibers. Fazeli et al. [28] conducted a study on the surface treatment of recycled carbon fibers using a combination of PUDs and silane compounds, exploring its impact on the mechanical properties of ensuing composites. The application of a flexible coating comprising PUDs crosslinked with silane coupling agents onto the recycled carbon fiber surface yielded noteworthy enhancements in impact, tensile, and flexural strengths in epoxy-based CFRPCs.

In addition to the use of linear PUDs, there is a growing interest in incorporating hyperbranched polymers into fiber-reinforced polymer composites (FRPCs) as a means to enhance interfacial properties. Hyperbranched polymers exhibit spherical dendritic structures with cavities and numerous terminal functional groups. These distinctive features allow hyperbranched polymers to contribute to both mechanical interlocking and chemical bonding between the polymer matrix and carbon fibers. The incorporation of hyperbranched polymers represents a versatile strategy for optimizing the interface in composite materials based on carbon or glass fibers, which are typically supplied with commercial sizings with limited information on their nature and functionality. Thus, the incorporation of HBPs may broaden the range of materials and methodologies available for advancing FRPC technology [29,30]. 

The incorporation of carbon nanomaterials into FRPCs, specifically on the fiber surface, has been reported as a feasible approach to enhance the mechanical properties of such composite structures [31]. For example, Zhang et al. [32] produced composites with dispersed graphene oxide (GO) layers directly integrated onto the surface of individual carbon fibers as part of the fiber sizing process. The incorporation of 5 wt% GO sheets in this manner led to significant improvements in the interfacial and tensile properties of the resulting CFRPCs, highlighting the potential of integrating GO into fiber sizing as an effective strategy. Xiong et al. [33] introduced a novel strategy for enhancing the interface and mechanical properties of CFRPCs by grafting GO onto carbon fibers with HBPs using thiol-ene click chemistry and a vinyl-terminated hyperbranched polyester. The tensile and flexural strengths of corresponding CFRPCs increased by 47.6% and 65.8%, respectively.

CFRPCs obtained from carbon fibers modified with graphene nanoplatelets (GNPs) have been shown to exhibit enhanced mechanical and thermal properties [34]. A solution comprising GNPs in acetone, along with a small amount of resin/hardener, was formulated as a spraying solution for modifying dry fabrics suitable for the vacuum-assisted resin transfer infusion (VARI) process. Through this method, GNP-reinforced FRPCs were successfully fabricated, with GNPs uniformly distributed in the interlaminar regions. Analyses revealed effective immobilization of GNPs on the surfaces of carbon fibers post-spray coating. Moreover, significant enhancements were observed in the mechanical properties and thermal conductivity of the resulting epoxy-based CFRPCs. Specifically, the incorporation of 0.3 wt% GNPs led to the highest levels of flexural strength and interlaminar shear strength. Other studies have explored different matrices beyond epoxy in conjunction with GNPs and GOs to enhance the mechanical properties of FRPCs. Li et al. [35] successfully enhanced the interfacial properties of CF/copoly(phthalazinone ether sulfone)s (PPBESs)-based composites by incorporating multi-scale hybrid carbon fiber/GO (CF/GO) reinforcements. An optimized GO loading of 0.5% with a homogeneous distribution of GO by coating the hybrid fiber surface led to significant improvements in the PPBES composite’s interlaminar shear strength, reaching 91.5 MPa, and flexural strength, reaching 1886 MPa. These enhancements represented increases of 16.0% and 24.1%, respectively, compared with the non-reinforced counterpart. Furthermore, a reduction in the interface debonding in CF/GO (0.5%) composites suggested superior interface adhesion due to the incorporation of GO into the interface. Choi et al. [36] investigated the influence of nanomaterials and fiber interface angles on the mode I fracture toughness of woven CFRPCs. Three types of carbon nanomaterials—COOH-functionalized short multi-walled carbon nanotubes (S-MWCNT-COOH), MWCNTs, and GNPs—were investigated. Specimens were fabricated using the hand lay-up method, comprising 12 woven carbon fiber fabrics with or without 1 wt% nanomaterials. The incorporation of nanomaterials led to a mode I fracture toughness exceeding that of pure CFRP. Notably, the utilization of GNPs demonstrated superior effectiveness in enhancing the fracture toughness compared with other nanomaterials. Costa et al. [37] reported the improvement of the tensile strength of a high-density polyethylene-based FRPC with natural fibers by incorporating GNPs into the matrix. An increase of over 20% in the Young’s modulus was achieved compared with the high-density polyethylene composite alone, reaching 1.63 ± 0.15 GPa.

Although the vast majority of the literature studies offer unique strategies for enhancing interfacial interactions between the matrix and the fiber surface in FRPCs by the incorporation of various nanoparticles into this interface for improved mechanical properties, it becomes increasingly difficult to demonstrate such enhancements and improvements, particularly in the tensile properties of high-performance FRPCs with tensile strength (>900 MPa) and Young’s modulus (>60 GPa) values that are notably high to start with. On the other hand, the individual use of PUDs as post-sizing agents and GNPs as reinforcing agents has been separately demonstrated to effectively tailor the interface of FRPCs in previous studies. However, the combined incorporation of carbon nanomaterials in the presence of chemically functional PUDs into the fiber–matrix interface of FRPCs remains an area worthy of investigation for potential synergistic effects in enhancing the mechanical properties of high-performance FRPCs.

In this manuscript, the fiber–matrix interface of a high-performance CFRPC structure was tailored with GNPs in the presence of a waterborne, multi-functional polyurethane in an effort to improve the tensile properties of corresponding CFRPCs. GNPs were incorporated into the fiber–matrix interface of CFRPCs in the presence of a waterborne, highly branched, multi-functional polyurethane dispersion (HBPUD). The in-house synthesized HBPUD, possessing both amine and silane terminal groups in the backbone, was designed to act as both a dispersing agent for GNPs in aqueous media during the spray deposition and a reactive sizing agent for covalent bridging between the carbon fiber, epoxy matrix, and GNPs at the interface of the corresponding composite structures upon curing. For this purpose, aqueous dispersions containing various ratios of HBPUDs and GNPs were introduced onto carbon fiber fabric surfaces via a novel ultrasonic spray deposition technique to ensure a fine distribution of GNP particles and homogeneous surface coverage, followed by the fabrication of prepreg laminates using hot melt epoxy films to obtain CFRPC plates by stacking them and curing in an autoclave. In a comprehensive examination, the effects of the presence of a multi-functional polyurethane layer at the interface, the ratio of the polyurethane to GNPs, and the overall GNP content per unit area of the carbon fiber fabric on the tensile strength and Young’s modulus of the corresponding CFRPC plates were systematically investigated.

## 2. Materials and Methods

### 2.1. Materials 

Hexamethylene diisocyanate (HDI), polyol of ethylene glycol/adipic acid/butane diol (M_n_ = 2000 g/mole, Desmophen 1652), was purchased from Covestro (previously Bayer MaterialScience AG, Leverkusen, Germany). Diethylenetriamine (DETA) and acetone (99.5%) were purchased from Aldrich Chemical Corporation (Milwaukee, WI, USA). The 3-isocyanatopropyltriethoxysilane (IPTES) was kindly donated by Momentive Performance Materials (Niskayuna, NY, USA). Sodium 2-[(2-aminoethyl) amino] ethanesulphonate (Vestamin A-95, AEAS) was kindly donated by Evonik Industries (Essen, Germany). Graphene nanoplatelets (purity: 99.9%) (GNPs) with 3 nm of thickness, 1.5 μ diameter, and 800 m^2^/g specific surface area were purchased from Nanografi (Ankara, Turkey) [38]. Twill weave carbon fiber fabric woven with 400 gsm DowAksa (Istanbul, Turkey) 12K yarns (TW400) and uncured epoxy resin films with 145 gsm were provided by KordSA (Pendik, Turkey). The 3M Scotch-Weld AF163-2K adhesive was used for bonding specimens for tensile tests. 

### 2.2. Synthesis of Functional HBPUDs

**Figure 1:**
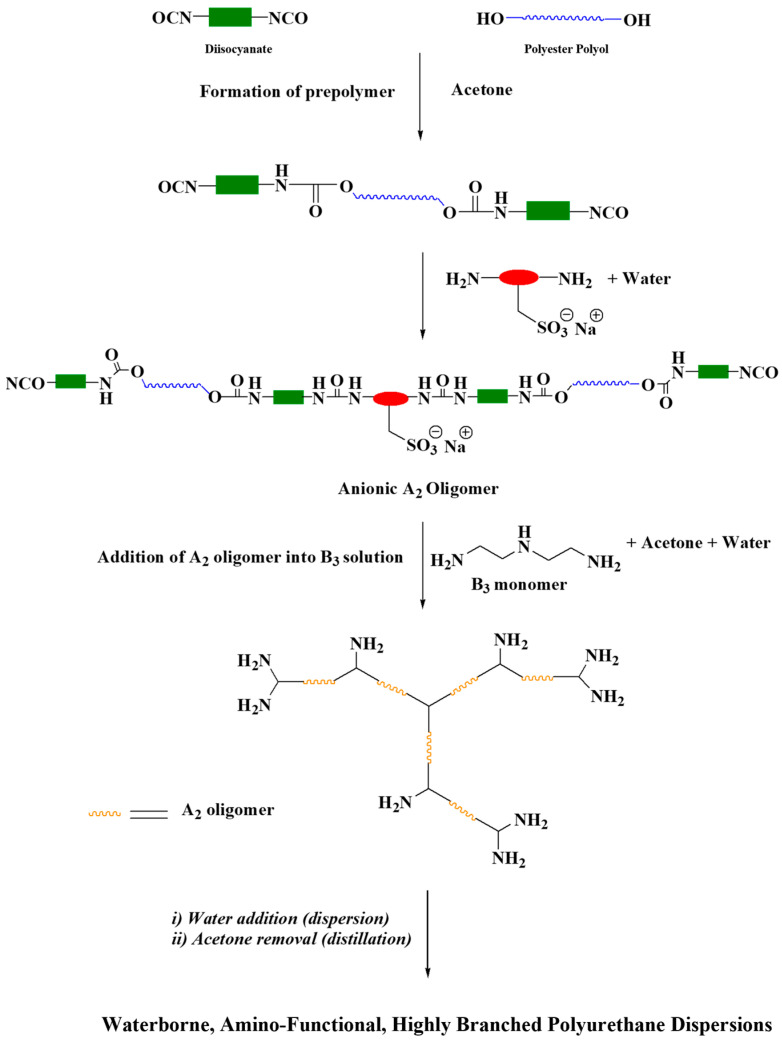
Synthesis of waterborne, amino-functional, highly branched PUDs via A_2_ + B_3_ approach (Green: HDI, Blue: polyol, Red: AEAS).

Waterborne HBPUD samples with (i) only amino- (HBPUD-0) and (ii) both amino- and silane-functional terminal groups (HBPUD-50) were designed and synthesized using the oligomeric A_2_ + B_3_ approach based on the chemical compositions given in Table 1 as previously reported [39]. The synthesis route (Figure 1) contained four steps: (a) preparation of the A_2_ oligomer, (b) branching by the reaction of the A_2_ oligomer with the B_3_ monomer, (c) functionalization, and (d) dispersion and distillation. A precalculated amount of polyester polyol (Table 1) was charged into the dried 3 L four-necked round-bottom flask equipped with an overhead stirrer, a reflux condenser, and a thermocouple that was connected to a heating mantle to control the reaction temperature. The polyol was dewatered by applying a vacuum (~2 mbar) for 15 min, at 75–85 °C. Upon the removal of the vacuum, the temperature was set to 60 °C, HDI was slowly added into the reaction flask, and the reaction was stirred for 3 h at 80 °C. The NCO content of the reaction mixture during the prepolymer process was monitored by the back-titration method [40], and the completion of the reaction was verified when the measured NCO content reached the theoretical NCO value. Upon the completion of the prepolymer reaction, the reaction temperature was set to 55 °C, and acetone was added to obtain the NCO-terminated polyurethane prepolymer with a concentration of 35–40 wt% in acetone at 48–50 °C. Following the prepolymer synthesis, 15% aqueous solution of a precalculated amount of the ionic monomer AEAS was fed dropwise into the reaction mixture at 48 °C to form the NCO-terminated, anionic A_2_ oligomer. The freshly prepared anionic A_2_ oligomer was then immediately transferred into an addition funnel, which was then slowly added into the preweighed DETA solution in acetone and water in a 5 L, four-necked round-bottom flask equipped with a reflux condenser, a mechanical stirrer, and a thermocouple. Upon the completion of the branching step by the slow addition of the ionic A2 oligomer into the B3 monomer solution, amino-functional, branched polyurethane was obtained in acetone. Next, distilled water was slowly added into the reaction mixture while vigorously stirring to disperse polyurethane chains in water while cooling the mixture to 42 °C. Finally, acetone was removed from the reaction mixture by vacuum distillation, and the complete removal of the acetone was ensured at 42 °C, 50 mbar. The final product, amino-functional HBPUD (denoted as HBPUD-0) with a solid content of 33 wt%, was collected by filtering through a 50-micron filtration medium.

**Figure 2:**
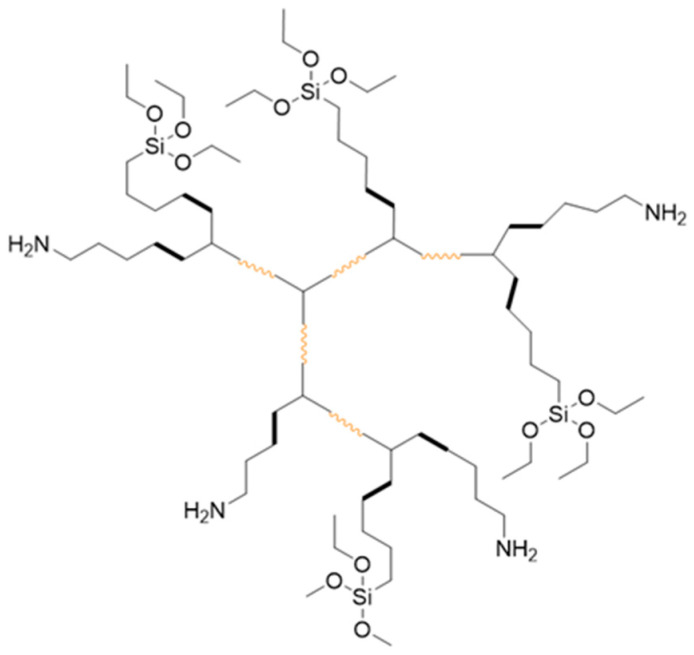
Representative structure of HBPUD-50.

In order to attain silane functionality and obtain HBPUDs with both amino- and silane-terminal groups, in a separate synthesis reaction, after the synthesis of amino-functional polyurethane in acetone as described above, a precalculated amount of IPTES compound was added dropwise into the reaction mixture immediately after the branching step at 48 °C to achieve a 50:50 ratio of amino–silane terminal groups, denoted as HBPUD-50. Figure 2 shows the structure and terminal groups of HBPUD-50. 

**Figure 3:**
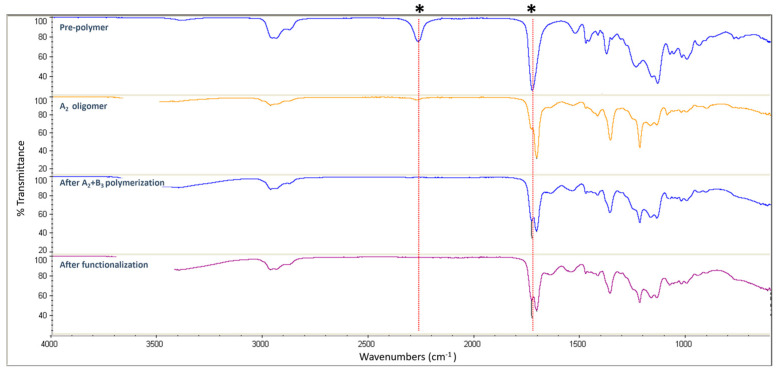
HBPUD-50 synthesis followed using FT-IR spectroscopy (*: bands corresponding to ~2260 and ~1725 cm^−1^ for NCO and C=O stretching vibrations, respectively).

FT-IR spectroscopy was used to monitor the functionalization step, by ensuring the absence of any NCO stretching vibration band (~2260 cm^−1^) from IPTES as shown in Figure 3. Upon the completion of the functionalization step, the dispersion and acetone distillation steps were applied as described above for the synthesis of amino-functional HBPUD (HBPUD-0). The chemical compositions of the synthesized HBPUD samples are given in Table 1.

### 2.3. Characterization of HBPUDs and PU Films

HBPUD syntheses were monitored, and final solid PU films were analyzed using a Nicolet IS10 Fourier Transform Infrared (FTIR) Spectrometer (Waltham, MA, USA) equipped with an ATR system with a 4 cm^−1^ resolution over 120 scans and ASTM D2572-97 back-titration method. 

The particle sizes and distributions of HBPUDs were determined using a ZetaSizer, Malvern Instruments (Malvern, UK), provided with laser diffraction and polarized light of three wavelength detectors. Approximately 0.1 mL of HBPUD was diluted with distilled water to an adequate concentration in the cell and measured at room temperature. The refractive indices of PU and water were 1.50 and 1.30, respectively. The HBPUD-50/GNP mixtures were analyzed using a Partica LA-960V2 (Horiba, Kyoto, Japan) wet circulation system equipped with a dispersant filling pump, liquid level sensor, circulation pump, 30 W in-line ultrasonic probe, and relief valve. The particle size analysis was conducted by gauging the angular deviation of light scattered by particles when traversing a laser beam. The machine employed the principles of Mie scattering and Fraunhofer diffraction to ascertain precise measurements [41]. 

Tensile stress–strain tests of dried polyurethane films were performed on a universal testing machine (Zwick Roell Z100 UTM, Ulm, Germany), with a load cell of 200 N, a crosshead speed of 25 mm/min, and a grip-to-grip separation of 22 mm, and dog bone-shaped samples were prepared and tested according to a standard test method [42]. Three to five specimens were measured, and their average stress–strain values with standard deviations were reported. 

Gel content (%) tests were carried out using Soxhlet extraction with toluene. For the gel content measurements, the dried polyurethane film (G1) and the thimble (G) were precisely weighed, and the polyurethane film was put into the thimble and extracted with toluene for 24 h. The thimble containing the film after the extraction was weighed again (G2) after drying. To calculate the gel content (%) of each polyurethane film, Equation (1) given below was used.

Gel content (%) = [(G2 − G)/G1] × 100(1)

Scanning electron microscopy (SEM) was employed using a Leo Supra 35VP FEG-SEM (Miami Beach, FL, USA) to examine the surface morphology of the dried HBPUD film samples, which were coated with gold and palladium for enhanced conductivity and imaging quality.

### 2.4. Preparation of Aqueous HBPUD/GNP Mixtures and Their Deposition onto Carbon Fiber Fabric Surfaces

**Figure 4:**
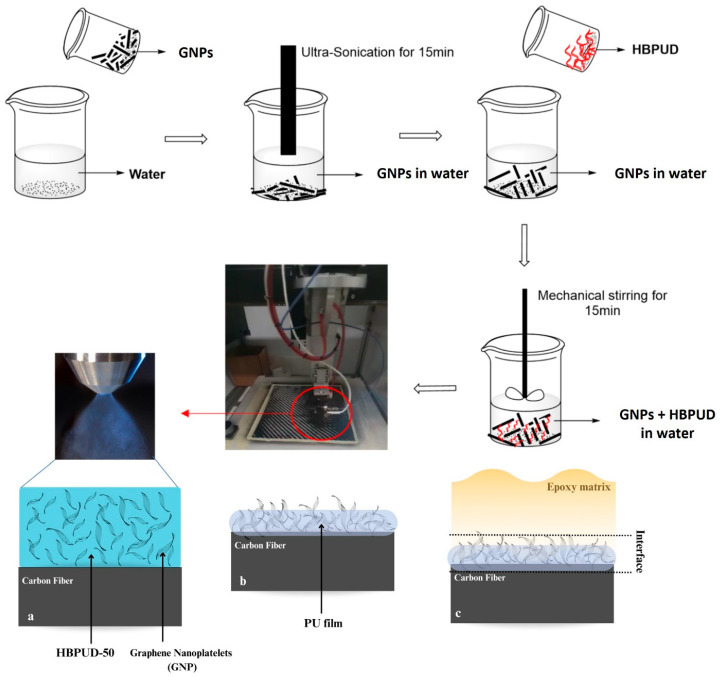
Preparation of HBPUD-50/GNP dispersions, their ultrasonic spray deposition onto the carbon fiber fabric surface, and the formation of the epoxy–carbon fiber interface containing PU and GNPs: (**a**) carbon fiber surface upon ultrasonic spray deposition, (**b**) carbon fiber surface upon drying, and (**c**) epoxy–carbon fiber interface in the final CFRPC.

The preparation of HBPUD-50/GNP mixtures in water and the deposition of this mixture onto the carbon fiber surface is schematically given in Figure 4. First, 0.25 g of GNPs was dispersed in 500 g of deionized water using probe ultrasonication (SONICA Q700 equipment, Niles, IL, USA) for 15 min under 70% amplitude with 5 s pulse on and 5 s pulse off. Then a predetermined amount of HBPUD-50 was mixed into this dispersion by mechanical stirring such that two different HBPUD-50/GNP mixtures were obtained with 0.05 wt% GNP concentration and solid PU:GNP weight ratios of 0.33:1 and 1:1. In addition, an aqueous dispersion of pure GNPs with 0.05 wt% and a pure HBPUD-50 dispersion with 0.05 wt% solid PU were prepared, which corresponded to 0:1 and 1:0 solid PU:GNP ratios, respectively.

Freshly prepared aqueous HBPUD-50/GNP mixtures were sprayed onto each side of 350 mm × 350 mm TW400 carbon fiber fabrics using a SONO-TEK Inc. (Milton, NY, USA), Flexi Coat ultrasonic spray coater with a 48 kHz, impact-type ultrasonic spray shaping nozzle. The spray was guided onto the substrate using jet air deflection aided by compressed air gas. At the end of the spray deposition, fabrics were dried on a heated plate at 50 °C for 1 day. The amount of GNPs deposited per unit surface area of carbon fiber fabric was controlled by the amount of HBPUD-50/GNP sample to be sprayed, such that 10, 20, and 30 mg of GNPs was deposited per m^2^ of each side of the carbon fiber fabric (denoted as 10, 20, and 30 mgsm) from each HBPUD-50/GNP sample, corresponding to 15 different carbon fiber fabrics in total, coated with HBPUD-50/GNP aqueous mixtures.

Samples of HBPUD-50/GNP mixtures with solid PU:GNP weight ratios of 0.33:1 and 1:1 dried in an oven overnight at 80 °C and pure GNPs (PU:GNP ratio of 0:1) were analyzed using a Nicolet iS50 FTIR Spectrometer (Waltham, MA, USA) in transmission mode by preparing KBr pellets containing approximately 0.05 wt% of each dried sample.

### 2.5. Fabrication of Prepregs and Manufacturing of CFRPC Plates

**Figure 5:**
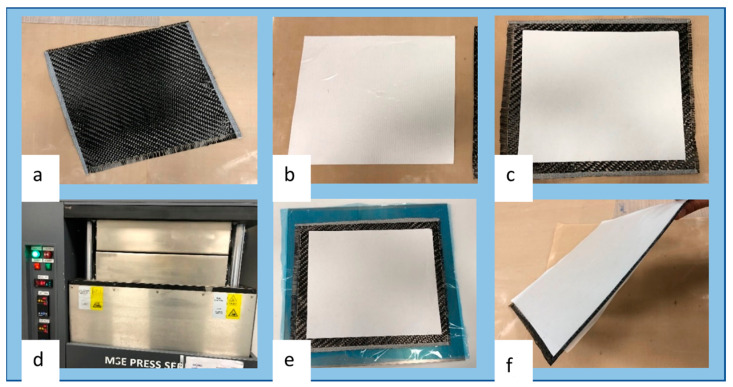
In-house prepreg laminate fabrication: (**a**) TW400 carbon fiber fabric (330 × 330 mm), (**b**) 145 gsm epoxy resin film (320 × 320 mm), (**c**) TW400 fabric sandwiched in between epoxy resin film, (**d**) resin impregnation in hot press, (**e**) prepreg (initial form out of hot press), and (**f**) prepreg after trimming the edges (300 × 300 mm, ready to lay up).

An in-house method was developed and used for the fabrication of prepreg materials from ultrasonic spray-coated carbon fiber fabrics and hot melt epoxy resin films utilizing a hot-press technique with optimized parameters. The detailed fabrication process and setup are illustrated in Figure 5, while specifications of the desired prepreg materials are detailed in Table 2, for which the production process was precisely tailored. 

TW400 fabric layers that were either as is or spray coated with each aqueous HBPUD-50/GNP mixture were cut into the precise dimensions of 330 mm × 330 mm (Figure 5a), and a roll of 145 gsm epoxy resin film was accurately cut into preforms with dimensions of 320 mm × 320 mm (Figure 5b) to ensure complete coverage without resin overflow. Concurrently, two layers of epoxy resin film were strategically placed on both surfaces of the TW400 carbon fiber fabric (Figure 5c) to promote an even resin distribution upon the application of pressure and heat. Each carbon fiber fabric sandwiched between epoxy resin films was placed in the hot press (Figure 5d) at 60 °C, under 0.1–0.2 ton-force pressure for 30–40 s ensuring the transformation of the sandwich structure into prepreg (Figure 5e). Last, prepregs out of the hot press were precisely trimmed to dimensions of 300 mm × 300 mm (Figure 5f), rendering them suitable for use in the manufacturing of CFRPC test plates. 

The carbon/epoxy prepregs prepared in house, as depicted in Figure 5f, were utilized in the manufacturing of CFRPC laminates from five layers of prepregs with stacking sequences of [0]_5s_ for tensile tests. Subsequently, stacked prepreg laminates were bagged for the autoclave manufacturing process with Teflon-coated glass fabrics (Fiberflon 828-25) on the autoclave trays, facilitating the plates’ removal post-curing and peel ply material application to both the top and bottom surfaces of all laminates. A vacuum blanket (Airtech N10) (Airtech, Huntington Beach, CA, USA) was then carefully placed over the laminates, and the assembly was sealed using leak-proof tape (AT 200Y) (Airtech) around the tray edges. Two vacuum valves were positioned diagonally on each tray, and the setup was enclosed with a vacuum bag (Airtech WL7400).

The autoclave curing cycle was conducted at 120 ± 3 °C, under a vacuum of −0.2 ± 0.05 bar and a positive pressure of 7 ± 0.2 bar, sustained for 1 h. The cooling phase ensued at a controlled rate of 3 ± 0.5 °C/min. Upon curing, the plates underwent thickness verification via the non-destructive A-Scan inspection method. Plates that passed this inspection that had thickness values of 2.30 ± 0.15 mm (fabricated from five layers of prepreg sheets with individual cured ply thicknesses of 0.46 ± 0.03 mm) were then subjected to the tab bonding process first, followed by the coupon cutting process. The tensile specimens were then accurately sectioned using a water jet milling system equipped with KUKA KR-16 Ultra F Robot (Kuka, Ausburg, Germany). 

A reference CFRPC sample denoted as CFRPC-Ref was manufactured using TW400 carbon fiber fabric as is, while CFRPC samples manufactured using prepregs from each spray-coated TW400 fabric were named as CFRPC-x-y:z, where x denoted the GNP deposition amount per meter square of TW400 fabric (mgsm), and y:z denoted the solid PU:GNP ratio in each spray deposition.

### 2.6. Characterization and Testing of CFRPC Plates

The surface morphologies of spray-coated carbon fiber samples and fractured CFRP sample surfaces were analyzed using a Leo SUPRA 35VP FEG-SEM. The images were taken at varying accelerating voltages between 2 kV and 5 kV using secondary electron imaging. 

The tensile properties of manufactured CFRPC plates were measured according to a test standard [46]. Test samples were prepared from each CFRPC plate with dimensions of 250 mm (length) × 15 mm (width). Tension test plates were tabbed with [+45°/−45°]_4s_ glass fiber-reinforced epoxy prepregs using 3M Scotch-Weld AF163-2K adhesive film (3M, St. Paul, MN, USA) and cured in the vacuum oven at 105 °C for 2 h. Tensile tests of the CFRPC plates were performed using INSTRON 5982 100 kN Universal Testing Systems (Norwood, MA, USA). A clip-on biaxial extensometer was initially attached to each tensile test specimen, which was removed before 0.5% strain during each test.

## 3. Results and Discussion

An anionic, isocyanate-terminated prepolymer was synthesized as an A_2_ oligomer, which was polymerized with DETA as the B_3_ monomer in dilute acetone solution to obtain highly branched, amino-functional polyurethane as shown in Figure 1. The polymerization was carried out in acetone medium, and the resulting polyurethane was dispersed in water to obtain waterborne, amino-functional HBPUDs as reported previously [39]. In this study, the reaction of amino-terminal groups with IPTES prior to the dispersion step enabled the partial conversion of amine terminal groups to silane groups as shown in Figure 2. The presence of both amine and silane terminal groups on the highly branched polyurethane backbone was envisioned to enhance interactions between the carbon fiber, GNPs, and the polymeric matrix both covalently and non-covalently when incorporated into the interface of CFRPCs. In this context, while silane terminal groups of the polyurethane were expected to react with residual hydroxyl groups present on the fiber and GNP surfaces, amine terminal units on the same polyurethane backbone were expected to react with the epoxy resin during the curing stage of the prepreg laminates. For this purpose, the HBPUD-50 sample was synthesized according to the composition given in Table 3, with an amine–silane terminal group molar ratio of 50:50. This sample was successfully obtained with a solid content of 33 wt% and an average particle size value of 84 nm (Table 3), which was stable over prolonged shelf storage.

**Figure 6:**
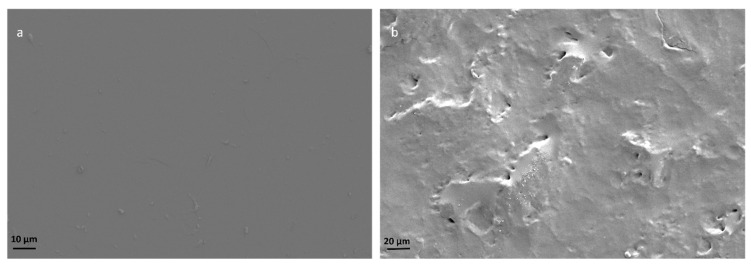
SEM images of the surfaces of solid polyurethane films from (**a**) HBPUD-0 and (**b**) HBPUD-50 samples.

The presence and the effect of silane terminal groups in the HBPUD-50 sample (Figure 2) were first evaluated in the pure polyurethane film as they were expected to lead to the self-crosslinking of the corresponding film upon casting and drying. SEM images of the surfaces of solid polyurethane films from the HBPUD-0 and HBPUD-50 samples are presented in Figure 6. Both samples formed continuous films. The amino-functional polyurethane had a smooth surface, yet it did not form a self-standing film with a mechanical integrity. The polyurethane film from the silane functional HBPUD-50 sample was self-standing, and its SEM images revealed a rougher surface with micro-voids, possibly due to the hydrolysis and self-condensation of silane terminal groups. 

**Figure 7:**
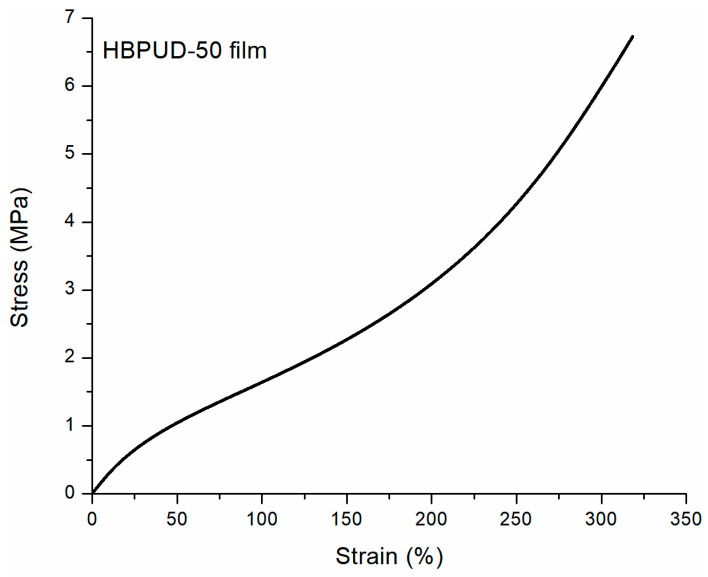
Tensile stress–strain curve of polyurethane film from HBPUD-50 sample.

**Figure 8:**
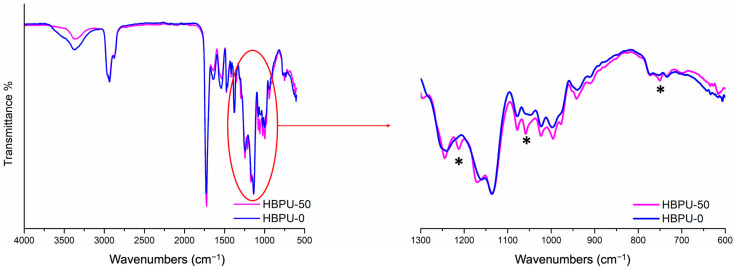
FT-IR spectra of dried PU films from HBPUD-0 and HBPUD-50 samples (*: bands corresponding to Si-O-Si at ~1200, 1050 and ~750 cm^−1^).

In order to assess the effects of silane terminal groups on the physical properties of the resulting polyurethane films, the gel content and tensile properties of standalone polyurethane films from HBPUD-0 and HBPUD-50 were compared as summarized in Table 3. While the HBPUD-0 sample resulted in a fully soluble film in toluene with 0 wt% gel content, the HBPUD-50 sample had >80 wt% gel content, which was attributed to the hydrolysis, self-condensation, and crosslinking of silane terminal groups (Figure S1) in the highly branched polyurethane backbone from the HBPUD-50 sample. While the HBPUD-0 sample did not form a self-standing film with mechanical integrity, the crosslinking mechanism resulted in self-standing polyurethane films from HBPUD-50 with the tensile stress–strain behavior shown in Figure 7 and tensile properties given in Table 3. Last, the presence of Si-O-Si groups in the polyurethane film from the HBPUD-50 sample was also verified by the peaks observed around 1200, 1050, and 750 cm^−1^ in the FT-IR spectrum of the film as shown in Figure 8.

Upon the synthesis and characterization of the HBPUD-50 sample, aqueous mixtures of GNPs and HBPUD-50 with different weight ratios were prepared for their deposition onto the carbon fiber fabric surface by ultrasonic spray deposition as depicted in Figure 4. The GNPs used in this study were formed of individual platelets with an average particle diameter of approximately 1.5 µm and a thickness less than 5 nm as previously analyzed using transmission electron microscopy (TEM) in the literature [47]. Considering the fact that these platelets were expected to form agglomerations rapidly in water, HBPUD was expected to enhance the stability and dispersibility of GNPs in water, which was a critical factor during their ultrasonic spray deposition onto the carbon fiber surface. As demonstrated in Figure S2, freshly prepared HBPUD-50/GNP mixtures with different weight ratios had relatively broad, uniform particle size distributions in water. Such broad distributions demonstrate the fact that GNPs are agglomerated in water, and ultrasonic spray deposition could play a key role in depositing them onto carbon fiber surfaces in smaller forms. When these mixtures were allowed to sit on the shelf for 24 h and shaken gently, an HBPUD-50/GNP mixture with a 1:1 solid PU:GNP weight ratio was observed to retain its original particle size distribution. The other two mixtures with less (0.33:1 ratio of solid PU:GNP) and no HBPUD-50 (0:1 ratio of solid PU:GNP) showed new, larger particle size shoulders, indicating that an adequate amount of HBPUD may assist in obtaining homogeneous, stable GNP dispersions in water. Dried samples of HBPUD-50/GNP mixtures were analyzed using FT-IR spectroscopy as shown in Figure S3. Pure GNPs (HBPUD-50/GNP 0:1) were characterized by a broad peak around 3400 cm^−1^ due to hydroxyl groups around the edges of the GNP sheets, small C–H stretching peaks below 3000 cm^−1^ presumably due to imperfections in the graphitic structure, and the main peak around 1615 cm^−1^ due to C=C bond stretching. On the other hand, pure polyurethane film was characterized by a strong C=O bond stretching peak around 1730 cm^−1^, along with C–H stretching peaks below 3000 cm^−1^ and a small peak arising from amine groups around 3300 cm^−1^. The FT-IR spectra of the HBPUD-50/GNP mixtures with 0.33:1 and 1:1 ratios of solid PU:GNP verified the presence of both polyurethane and GNPs in these mixtures by the presence of C=C bonds arising from GNPs and both C=O and C–H bonds increasing parallel with the polyurethane content.

**Figure 9:**
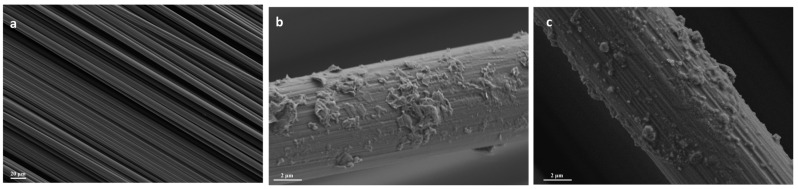
SEM images of (**a**) pristine carbon fiber, (**b**) fiber coated with 20 mgsm GNPs only, and (**c**) fiber coated with HBPUD/GNP-1:1 with 20 mgsm GNP deposition by ultrasonic spray deposition.

After the preparation of the HBPUD-50/GNP mixtures with different solid PU:GNP ratios, they were introduced onto carbon fiber fabric surfaces using a novel ultrasonic spray deposition method as demonstrated in Figure 4. The ultrasonic shaping nozzle of the spray equipment was expected to break up the agglomerates of GNPs in the aqueous medium immediately prior to the deposition of GNPs and enable uniform distribution of them on the coated carbon fiber surface. In this study, HBPUD-50/GNP mixtures with 0:1, 0.33:1, 1:1, and 1:0 ratios of solid PU:GNP were sprayed onto each side of 350 mm × 350 mm carbon fiber fabrics by ultrasonic spray deposition. While the samples with 0:1 and 1:0 weight ratios of PU:GNP corresponded to the deposition of pure GNPs and pure PU, respectively, samples with 0.33:1 and 1:1 weight ratios allowed the investigation of the presence of both PU and GNPs with two different ratios at the fiber–matrix interface. Each HBPUD-50/GNP mixture, as well as the pure HBPUD-50 (1:0 ratio) and GNP dispersion (0:1 ratio), was spray deposited in specific amounts to achieve depositions of 10, 20, and 30 mg of GNPs per m^2^ (mgsm) of each side of the carbon fiber fabric. The amount of the pure HBPUD-50 sample was adjusted to deposit a solid PU amount the same as that of the 1:1 solid PU:GNP sample for each mgsm deposition series. It should be noted that the depositions of 10, 20, and 30 mgsm GNPs corresponded to approximately 0.003, 0.006, and 0.009 wt% GNPs in the overall composite structure, respectively, when calculated based on the average areal weight of each prepreg sheet given in Table 2. Upon the spray deposition of HBPUD-50/GNP mixtures with different ratios in each deposition series, each sprayed carbon fiber fabric sample underwent an overnight drying process, during which water was removed and a nanocomposite film layer was formed by facilitating the self-crosslinking or reaction of silane terminal groups with GNP and carbon fiber surfaces. SEM images of the uncoated carbon fiber surface and the ones coated with 20 mgsm GNP from pure GNP dispersion and from HBPUD-50/GNP mixtures with 1:1 PU:GNP ratios are shown in Figure 9. The successful deposition of pure GNPs onto the originally smooth carbon fiber surfaces is visible in Figure 9b, showing a significant change in the surface morphology of fibers with the aid of the ultrasonic spray deposition. Visually, the surface of fibers coated with the HBPUD-50/GNP sample (with a 1:1 ratio of solid PU:GNP) appears similar to that of the pure GNP-coated one (Figure 9c), while a better attachment of GNP particles onto the fiber surface is expected due to the presence of a polyurethane layer, although it is not visible in the SEM images. A chemical bond is expected to develop between silane terminal groups of polyurethane and GNP or carbon fiber surfaces while retaining amine terminal groups, resulting in the establishment of an intricate interface between the fiber and the matrix to be introduced. 

Following the ultrasonic spray deposition of each HBPUD-50/GNP, as well as pure HBPUD-50 and GNP dispersions onto carbon fiber fabrics with 10, 20, and 30 mgsm GNP depositions from each dispersion, prepreg laminates were fabricated by sandwiching each carbon fiber fabric in between epoxy resin films with standard gsm values. The optimized parameters used in the in-house process depicted in Figure 5 ensured the robust adhesion of the resin to the fiber without resin overflow, while maintaining the fiber fabric’s integrity without causing damage, ensuring the transformation of the sandwich structure into prepreg form. The carbon/epoxy prepregs prepared in house were utilized in the manufacturing of CFRPC test plates by stacking laminates in [0]_5s_ orientation, followed by autoclave curing in vacuum bags. Fifteen different CFRPC test plates, each formed of five prepreg layers, with varying GNP amounts or PU:GNP ratios, were manufactured, along with a reference CFRPC manufactured from TW400 carbon fiber fabric without any HBPUD-50 and/or GNP deposition.

**Figure 10:**
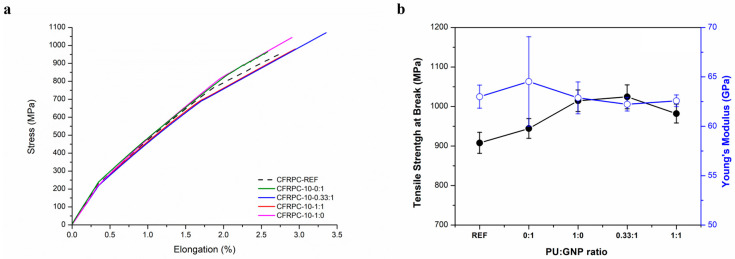
(**a**) Tensile stress–strain curves of CFRPC-10 test plate series and (**b**) variation in tensile properties of CFRPC-10 series as a function of PU:GNP ratio.

The fabrication and testing of the CFRPC series with 10, 20, and 30 mgsm GNP deposition on the carbon fiber surface allowed a systematic investigation of the influence of varying the content of GNPs and/or solid PU at the fiber–matrix interface on the tensile properties of CFRPCs. In Figure 10a, representative stress–strain curves of the CFPRC-10 series are shown, while Figure 10b displays the variation of the average tensile strength at break and the Young’s modulus values of the CFRPC samples with a GNP content of 10 mgsm and varying PU:GNP ratios at the interface. The deposition of 10 mgsm GNPs in the CFRPC-10-0:1 sample resulted in a slight increase in the tensile strength and modulus; however, a relatively large standard deviation especially in the modulus value indicated that GNPs alone may not have been homogeneously distributed at the interface. On the other hand, by the incorporation of 10 mgsm PU only from HBPUD-50, the CFRPC-10-1:0 sample showed an approximately 12% increase in the tensile strength reaching 1014.6 MPa, albeit with a slight decrease in the Young’s modulus value. Furthermore, the CFRPC-10-0.33:1 with both PU and GNPs showed similar tensile properties to those of the CFRPC-10-1:0 sample, whereas an increased amount of PU in the HBPUD-50/GNP mixture corresponding to the CFRPC-10-1:1 sample resulted in a slight decrease in the tensile strength value reaching 982.2 MPa, still remaining above the reference CFRPC. In conclusion, although a clear trend was not observed as a function of the PU:GNP ratio, the incorporation of PU only or GNPs in the presence of PU resulted in increased tensile strength values with no change in the Young’s modulus.

**Figure 11:**
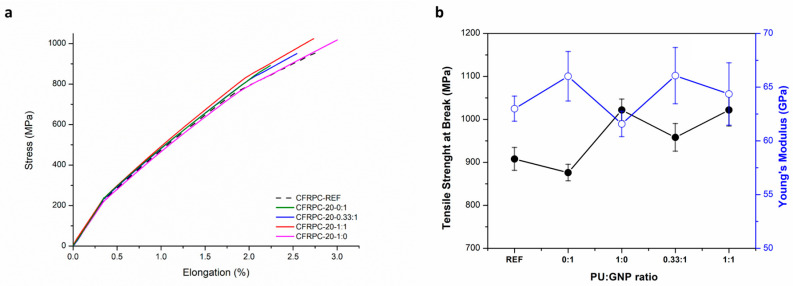
(**a**) Tensile stress–strain curves of CFRPC-20 series and (**b**) variation in tensile properties of CFRPC-20 series as a function of PU:GNP ratio.

Stress–strain curves of the CFRPC-20 series with 20 mgsm GNPs deposited alone or in the presence of HBPUD-50 are plotted in Figure 11a, and the variation in tensile properties as a function of PU:GNP ratios is given in Figure 11b. The increased amount of incorporated GNPs from 10 mgsm to 20 mgsm resulted in a significantly increased Young’s modulus but reduced tensile strength compared with the reference CFRPC sample. This suggested that a certain amount of GNPs at the interface without any attachment purely contributed to an increase in the modulus values. On the other hand, the incorporation of 20 mgsm PU only from HBPUD-50 in CFRPC-20-1:0 resulted in a significantly increased tensile strength value reaching above 1000 MPa and a slightly decreased Young’s modulus value compared with both the CFRPC-Ref and CFRPC-20-0:1 samples. Interestingly, the incorporation of a combination of PU and GNPs at weight ratios of 0.33:1 and 1:1 resulted in a synergistic effect. In the CFRPC sample having a combination of PU and GNPs at a weight ratio of 0.33:1 (CFRPC-20-0.33:1), the tensile strength value was moderately increased above 950 MPa, while the Young’s modulus value remained similar to that of the 20 mgsm pure GNPs incorporated CFRPC sample (CFRPC-20-0:1). In the case of the CFRPC-20-1:1 sample with increased PU content in combination with GNPs, the tensile strength value further increased compared with the CFRPC-20-0.33:1 sample, reaching the tensile strength value of the CFRPC-20-1:0 sample with pure PU, with a Young’s modulus value in between those of the CFRPC-pure and CFRPC-20-0:1 samples.

**Figure 12:**
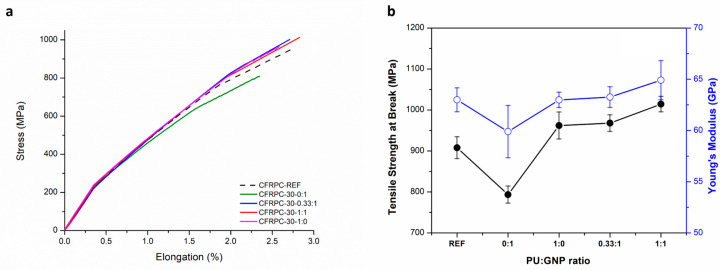
(**a**) Tensile stress–strain curves of CFRPC-30 series and (**b**) variation in tensile properties of CFRPC-30 series as a function of PU:GNP ratio.

Figure 12 shows the tensile properties of CFRPC samples with 30 mgsm GNPs at the interface alone or in combination with amine and silane functional polyurethane. The increased content of pure GNPs at the fiber–matrix interface resulted in a drastic decrease in not only the tensile strength but also the Young’s modulus value, contrary to the 20 mgsm pure GNPs incorporated CFRPC sample. On the other hand, 30 mgsm incorporation of only PU at the interface showed a slight improvement in the tensile strength without any changes in the Young’s modulus compared with the reference CFRPC. The incorporation of a combination of PU and GNPs at different weight ratios resulted in a visible trend of improved tensile strength and Young’s modulus behavior such that while the CFRPC-30-0.33:1 sample was similar to the CFRPC-30-1:0 sample with only PU at the interface, the CFRPC-30-1:1 sample containing equivalent weights of solid PU and GNPs stood out among all samples with a significantly improved average tensile strength value above 1000 MPa and a moderately increased Young’s modulus value around 65 MPa. It should be noted that our preliminary studies on increasing the incorporated GNP and/or PU content beyond 30 mgsm did not show any significant changes in the mechanical properties of the corresponding CFRPC laminates. Yet, the incorporation of high amounts of GNPs or other nanomaterials onto fiber fabric surfaces by ultrasonic spray deposition can be a promising approach in improving the thermal or electrical conductivity of FRPCs. 

A comprehensive analysis of the tensile behavior of all samples clearly indicated that the incorporation of relatively rigid GNPs alone into the interface without any attachment or chemical interactions solely improved the stiffness of corresponding samples up to 20 mgsm GNP incorporation, above which all tensile properties significantly decreased presumably due to an agglomeration effect of GNPs. On the other hand, the incorporation of a chemically functional PU layer alone into the fiber–matrix interface resulted in the improvement of mechanical properties through the enhancement of interfacial interactions, which was reflected as a significant increase in the tensile properties and clearly evidenced in the stress–strain curves of corresponding samples. In the case of the combined use of GNPs and a functional PU, a stiffening effect with the aid of GNPs and enhancement of interfacial interactions with the aid of a multi-functional PU layer through chemical bonding and interactions resulted in the improvement of tensile strength while maintaining or improving the initial Young’s modulus with the optimum content of PU and GNPs, such as in the CFRPC-20 series. 

**Figure 13:**
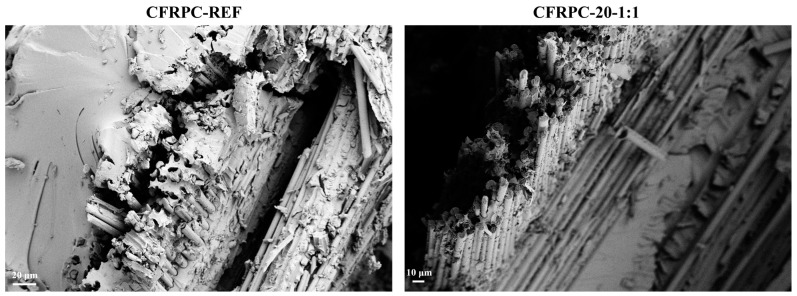
SEM images of a cross-section of the fractured surface of CFRPC-REF and CFRPC-20-1:1 samples.

The presented enhancement of interfacial interactions with the use of GNPs and multi-functional PU has been further assessed by SEM analysis of selected CFRPC samples after fracture. As illustrated in Figure 13, the reference CFRPC sample’s failure occurred predominantly through progressive interfacial debonding and fiber pullout, leading to arbitrary fiber breakage at multiple levels along the fiber direction and voids in the matrix. In contrast, when one of the best performing CFRPC samples’ (CFRPC-20-1:1) fractured surface was analyzed, the interface between the fiber and matrix remained almost intact after the failure, showing fewer fiber pullouts and more uniform fiber breakage, which provided evidence of strong interfacial bonding and contribution to improved tensile properties. 

Here, we demonstrated a novel approach to enhance the interfacial interactions and improve the tensile properties of fiber-reinforced polymer composites (FRPCs) by combining graphene nanoplatelets (GNPs) and a multi-functional polyurethane at the fiber–matrix interface using ultrasonic spray deposition. This method resulted in significant improvements in the tensile properties of FRPCs with as little as 20 to 30 mg of GNPs and PU/m^2^ of carbon fiber fabric, corresponding to approximately 0.006 to 0.009 wt% of each component in the overall composite structure. Notably, our study achieved these improvements with much lower amounts of carbon nanomaterials compared with previous studies. For instance, a prior study with a similar approach and composition of composite structure reported a notable increase in the tensile strength of CFRPCs from approximately 700 MPa to 850 MPa with the interfacial incorporation of 0.3 wt% GNPs, which is over 30 times higher than the GNP content used in our study [34]. It is important to point out that the waterborne, multi-functional polyurethane described in our study shows promise as a chemical compatibilizer and sizing agent, potentially enhancing interfacial interactions between dissimilar surfaces in composite materials synergistically when combined with various nanoparticles.

## 4. Conclusions

The incorporation of GNPs in the presence of a multi-functional polyurethane via their deposition onto the carbon fabric surface from an aqueous dispersion using an ultrasonic spraying deposition technique was systematically investigated. The synthesis and characterization of HBPUDs possessing only amine or both amine and silane terminal groups were carried out, aiming to understand the role of silane terminal groups on the polyurethane backbone. The presence of silane terminal groups on the polyurethane backbone led to a very high gel content and decent mechanical properties in resulting films from the HBPUD-50 sample, whereas the solid PU film from the HBPUD-0 sample with only amine terminal groups did not form a self-standing film. The subsequent preparation of GNPs with HBPUD-50 at different ratios assisted in keeping a homogeneous dispersion for the ultrasonic spray deposition of these mixtures onto carbon fiber fabrics to incorporate both GNPs and multi-functional polyurethane chains at the fiber–matrix interface of CFRPCs by the preparation of their prepreg laminates, stacking, and autoclave curing. A systematic study on the relative content of GNPs and the PU:GNP weight ratio at the fiber–matrix interface showed that a synergistic effect of both stiffening and enhancement of interfacial interactions was achieved, resulting in the improvement of the tensile strength values from approximately 908 MPa up to 1022 MPa and Young’s modulus values from 63 MPa up to 66 MPa. This study underscored the importance of carefully tuning the GNP content and PU:GNP ratio in tailoring these tensile properties in high-performance CFRPCs. 

## Figures and Tables

**Table 1:** Chemical compositions of synthesized HBPUD samples.

Component	Sample Name	
	HBPUD-0	HBPUD-50
Polyester polyol (g)	911.60	911.60
Diisocyanate (g)	144.62	144.65
AEAS (g)	109.74	109.74
DETA (g)	21.33	21.33
IPTES (g)	0	37.32

**Table 2:** Specifications of desired prepreg materials.

Test Standard	Test Result Type	Desired Range	Average Result	Unit
[43]	Prepreg areal weight (PAW)	690 ± 27	708	g/m^2^
[43]	Fiber areal weight (FAW)	400 ± 12	411	g/m^2^
[44]	Glass transition temperature (T_g_)	−3.5 ± 3.5	−1.4	°C
[44]	Curing enthalpy (ΔH)	100 ± 50	81	(J/g)
[45]	Gel time	15 ± 5	15	min

**Table 3:** Physical and mechanical properties of HBPU-0 and HBPU-50 (*: HBPUD-0 sample did not form a self-standing film).

	Sample Name	
	HBPUD-0	HBPUD-50
Particle size of dispersion (nm)	80	84
*Film properties*		
Tensile strength at break (MPa)	*	6.1 ± 1.1
Elongation at break (%)	*	301.2 ± 15.6
Young’s modulus (MPa)	*	3.2 ± 0.6
Gel content of film (wt%)	0	84

## Data Availability

Data are contained within the article and Supplementary Materials.

## Supplementary Materials

The following supporting information can be downloaded at https://www.mdpi.com/article/10.3390/polym16060828/s1: Figure S1: Self-crosslinking mechanism of HBPUD-50 upon film formation; Figure S2: Particle size analyses of HBPUD-50/GNP dispersion mixtures (a) freshly prepared and (b) gently shaken after one day of storage (all measurements were performed at 0.05 wt% concentration); Figure S3: FT-IR spectra of dried HBPUD-50/GNP samples.
