# Supplementary material for: Incorporation of Graphene Nanoplatelets into Fiber-Reinforced Polymer Composites in the Presence of Highly Branched Waterborne Polyurethanes
Source: Polymers (Basel). 2024 Mar 16;16(6):828. doi: 10.3390/polym16060828 (PMC10975395; doi:10.3390/polym16060828)
Supplement: Supplementary file 1 [file polymers-16-00828-s001.zip › polymers-2867876-supplementary.pdf]

Supporting Information

Incorporation of Graphene Nanoplatelets into Fiber-Reinforced Polymeric Composites in the Presence of Highly Branched Waterborne Polyurethanes

Ayşe Durmuş-Sayar[1,2], Murat Tansan[1], Tuğçe Çinko-Çoban[1], Dilay Serttan[1], Bekir Dizman[1,2], Mehmet Yildiz[1,2], Serkan Ünal [1,2,*]

[1] Sabanci University Integrated Manufacturing Technologies Research and Application Center & Composite Technologies Centre of Excellence, Teknopark Istanbul, 34906, Pendik, Istanbul, Turkey

[2] Faculty of Engineering and Natural Sciences, Sabanci University, Tuzla, 34956, Istanbul, Turkey

Corresponding author: Email address: serkan.unal@sabanciuniv.edu

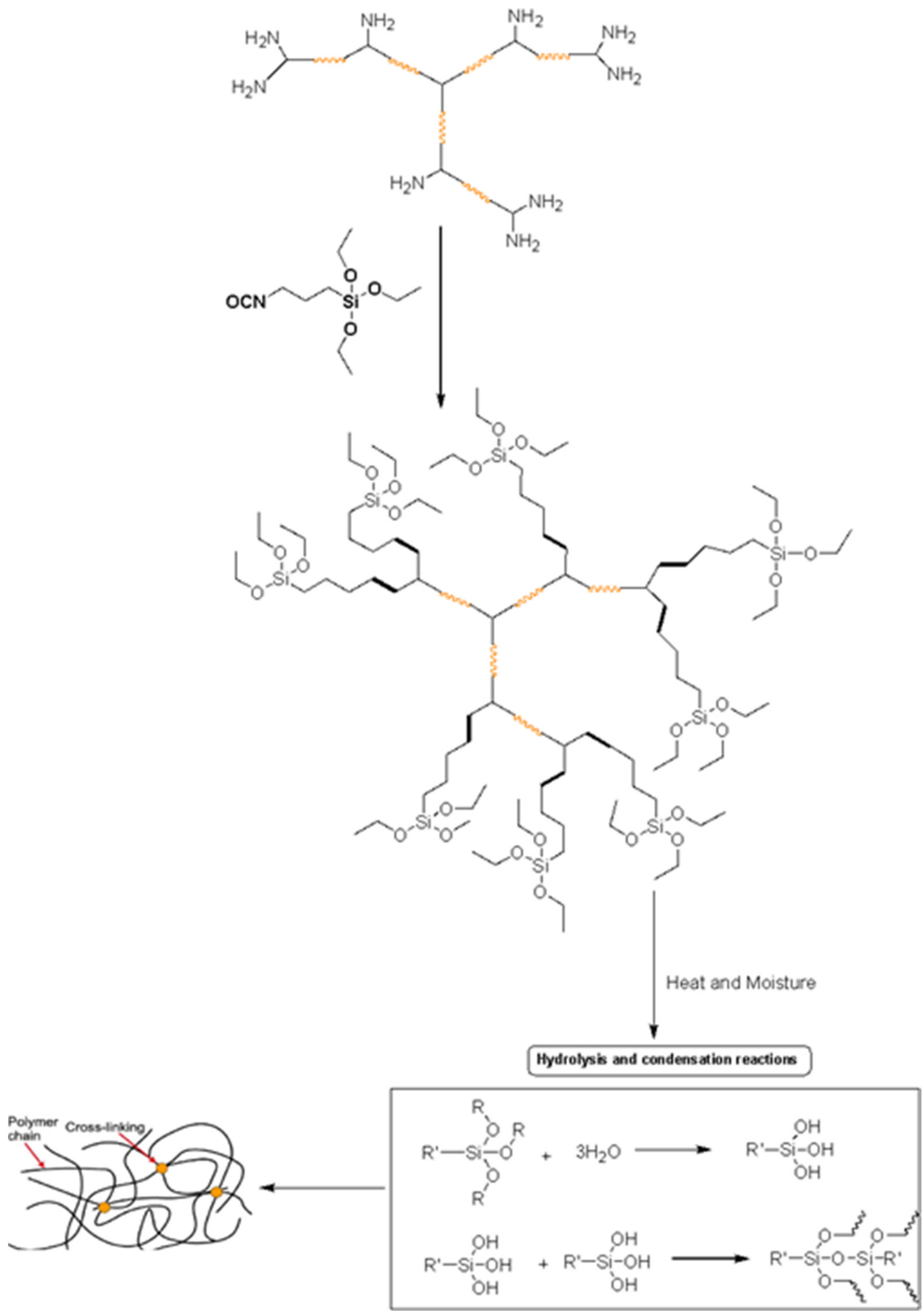

Figure S1. Self-crosslinking mechanism of HBPU-50 upon film formation.

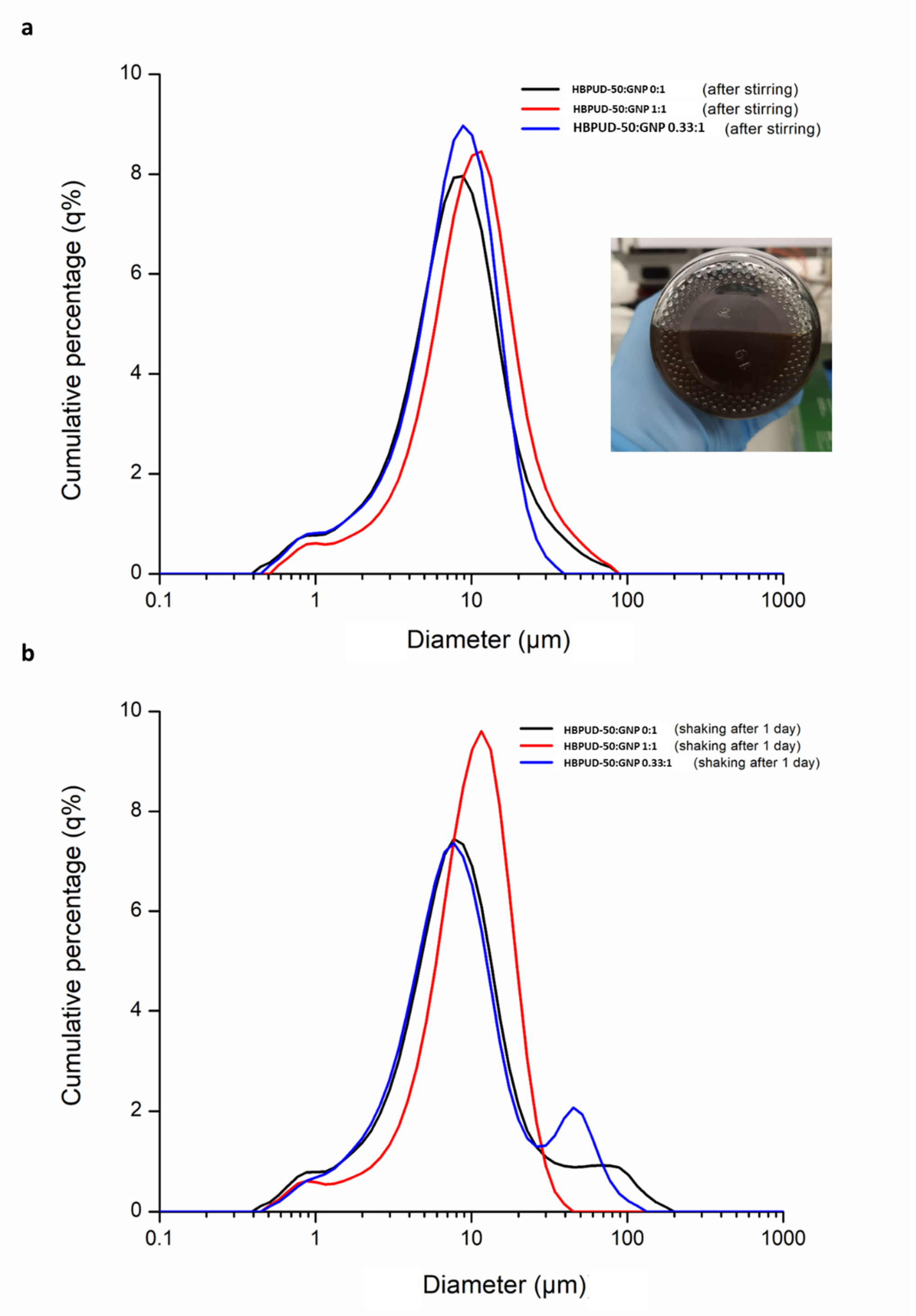

Figure S2. Particle size analyses of HBPUD-50/GNP dispersion mixtures are a) freshly prepared, and b) gently shaken after one day of storage (All measurements were performed at 0.05wt.% concentration).

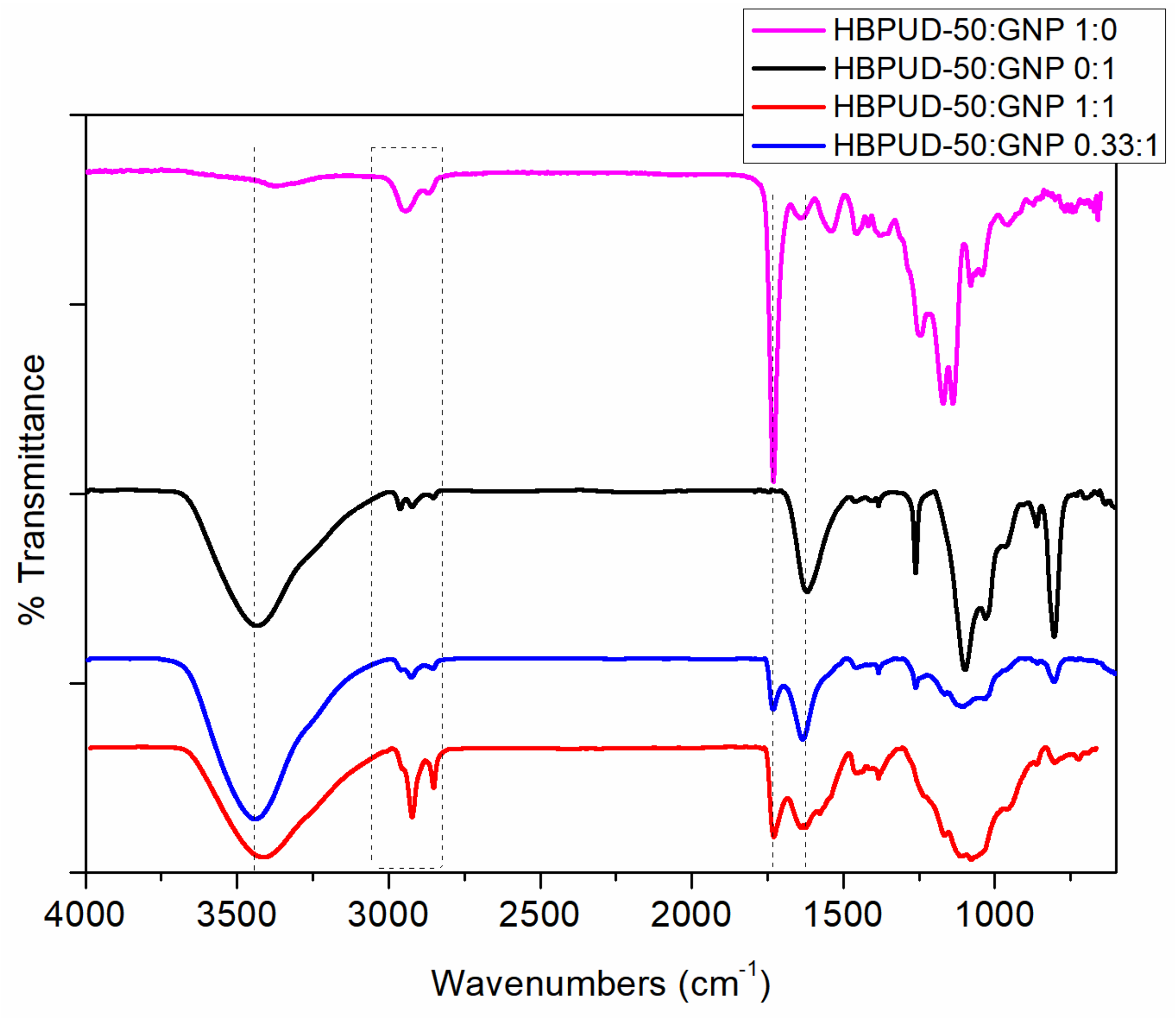

Figure S3. FT-IR spectra of dried HBPUD-50/GNP samples
